# Structural Instability of Epitaxial (001) BiFeO_3_ Thin Films under Tensile Strain

**DOI:** 10.1038/srep04631

**Published:** 2014-04-10

**Authors:** Zhen Fan, John Wang, Michael B. Sullivan, Alfred Huan, David J. Singh, Khuong P. Ong

**Affiliations:** 1 Department of Materials Science and Engineering, National University of Singapore, 9 Engineering Drive 1, 117576, Singapore; 2 Institute of High Performance Computing, Agency of Science, Technology and Research (A*STAR), 1 Fusionopolis Way, 138632, Singapore; 3 Materials Science and Technology Division, Oak Ridge National Laboratory, Oak Ridge, Tennessee 37831-6056, USA

## Abstract

We explore BiFeO_3_ under tensile strain using first-principles calculations. We find that the actual structures are more complex than what had been previously thought, and that there is a strong shear deformation type structural instability which modifies the properties. Specifically, we find that normal tensile strain leads to structural instabilities with a large induced shear deformation in (001) BiFeO_3_ thin films. These induced shear deformations in (001) BiFeO_3_ thin films under tension stabilize the (001) BiFeO_3_ thin films and lead to C*c* and I*ma2* phases that are more stable than the P*mc2_1_* phase at high tensile strain. The induced shear deformation shifts the C*c* to I*ma2* phase transition towards lower tensile strain region (~1% less), prevents monoclinic tilt and oxygen octahedral tilts, and increases the ferroelectric polarization. The induced shear deformation also strongly affects the electronic structure. The results are discussed in relation to growth of BiFeO_3_ thin films on cubic and tetragonal substrates involving high levels of tensile strain.

The interplay between the structure and bonding of materials and their physical properties is at the core of condensed matter physics. Bonding sets the electronic structure of a material and both controls and depends on the crystal structure. While this affects all physical properties, the interplay of bonding with ferroelectricity and magnetism are particularly delicate and interesting. Ferroelectricity may be regarded as a polar lattice instability that arises because of poorly satisfied bonding, while magnetism and magnetic interactions depend strongly on bond lengths and bond angles, e.g. through hopping integrals that control superexchange. For this reason, strain engineering of oxide ferroelectrics and magnetoelectrics through epitaxial growth has been a particularly effective approach for realizing new properties^1^,^2^,^3^,^4^,^5^,^6^,^7^,^8^.

BiFeO_3_ (BFO) is a particularly interesting material from this point of view. It can be epitaxially grown on a variety of oxide substrates with an exceptionally large range of achievable strain, and this leads to a wide range of properties and structures. For example, the observed conversion of a rhombohedral ferroelectric ground state to a super-tetragonal ferroelectric with giant polarization implies large piezoelectric couplings^9^,^10^. The large range of strain and resulting diverse properties attainable in BFO films is presumably a consequence of the interplay of the strong ferroelectricity driven by the lone pairs on Bi with the large octahedral tilts. BFO is of interest also for applications in data storage related to the large polarization, potentially magnetoelectrics (the material is antiferromagnetic but the magnetic interactions are strongly coupled to the structure), and in other areas^11^,^12^,^13^,^14^,^15^,^16^. Large realizable strain and its coupling to competing structural orders suggest complex behavior and raise the question of whether there are new states that are attainable but have not yet been identified. Here, we explore BFO under tensile strain and find that the actual structures are more complex than what had been previously thought, and that there is a strong shear deformation type structural instability that modifies the properties from what had been supposed.

Bulk BFO shows a rhombohedral symmetry with space group R*3c* and lattice parameters of a = 3.96 Å and α = 89.4°^17^. The spontaneous polarization in bulk BFO is along a pseudocubic [111] direction and reaches a large value of 90 ~ 100 μC/cm^2^ 18,19. As mentioned, strained epitaxial BFO films can be grown on different substrates and this can stabilize different phases and enable tuning of the physical properties^7^. So far, most reported epitaxial BFO thin films have been grown on substrates with compressive strain^9^,^20^,^21^. Theoretical and experimental studies show that compressive strain induces successive Rhombohedral (R) - Monoclinic (M_A_) - Monoclinic (M_C_) - Tetragonal (T) phase transitions in BFO (001) thin films accompanied by changes in the magnitude and orientation of ferroelectric polarization^22^,^23^. According to first-principles calculations, a fully tetragonal phase with giant c/a ratio ~1.26 and a very large polarization of ~150 μC/cm^2^ 24,25 can be obtained at a very high level of compressive strain exceeding −4.5%. Experimentally, a high growth rate was shown to stabilize the tetragonal phase, even at lower strain levels, specifically when BFO is deposited on SrTiO_3_ substrates at a smaller compressive strain of approximately −1.5%^25^,^26^. Turning to tensile strain, it was reported that a modest tensile strain could lead to a monoclinic M_B_ phase and a slight rotation in the polarization from [111] direction^27^. Dupe et al. predicted that under a tensile strain of ~8%, BFO thin films would undergo a first-order phase transition from the monoclinic space group C*c* to an orthorhombic I*ma2* structure^5^. Their work also indicates that tensile strain can affect the direction and magnitude of the magnetization and modifies certain magnetoelectric coefficients. However, Yang et al., based on epitaxial condition (***a_1_***, ***a_2_***, ***a_3_***) ~ (*2*, *2*, *2*)*a_IP_*, (*a_IP_* is the inplane lattice constant), found that for tensile strains above 5%, a different P*mc2_1_* phase is more stable than the C*c* and I*ma2* phases^6^.

Our initial motivation was that while both compressive strain and tensile strain have been investigated for epitaxial BFO thin films, the influence of shear deformation on BFO was not studied even though the coupling of strain to physical properties suggests that new phenomena may be observed. Experimentally, it is difficult to study deformations (from now on deformation means shear deformation) because of the limited choices of substrates. Nonetheless, when growing rhombic (001) BFO (in-plane angle 89.4°) on typical cubic or orthorhombic substrates (in-plane angle 90°), a deformation is introduced^28^,^29^. However, it is quite small and usually assumed to be zero^28^. It has been reported that deformations in ferroelectric BaTiO_3_ and PbTiO_3_ thin films can have an impact on the equilibrium phases, polarization and dielectric properties^30^,^31^,^32^. We find that under tensile strain, a deformation is spontaneously generated in otherwise unconstrained (001) BFO thin films and it has a strong impact on the detailed structure and ferroelectric polarization. We also revisited the stability of phases C*c*, I*ma2* and P*mc2_1_* (without influence of deformation) focusing on the stability of P*mc2_1_* structure and find quantitative differences from prior reports particularly regarding the stability of the orthorhombic phase; in particular, it is less stable than previously reported even without deformation.

## Results

### Structural instability of (001) BiFeO_3_ thin films under tensile strain

**Figure 1:**
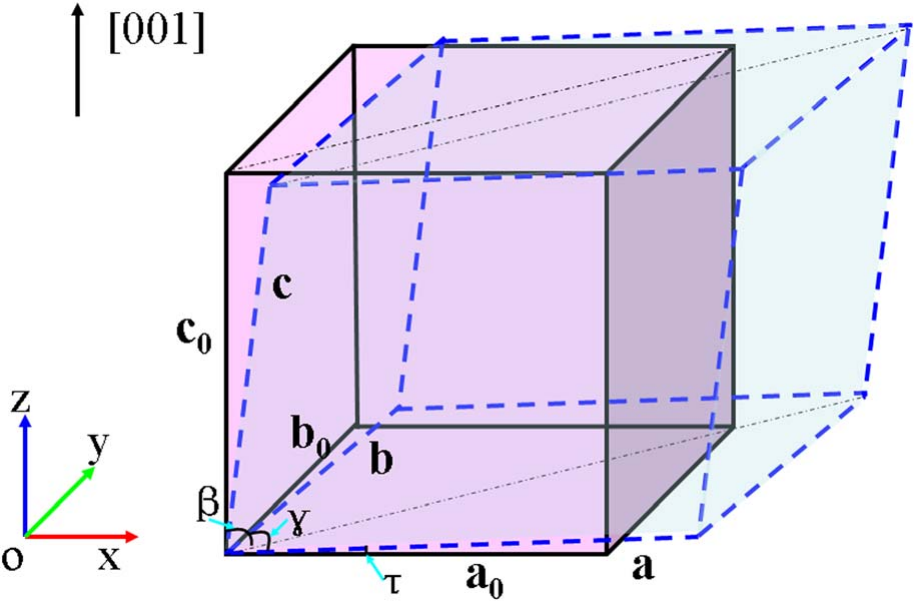
Schematic of the orientation of Cartesian coordinates and the constrained structure (dashed line) in relation to the pseudo-cubic structure (solid line).

**Figure 2:**
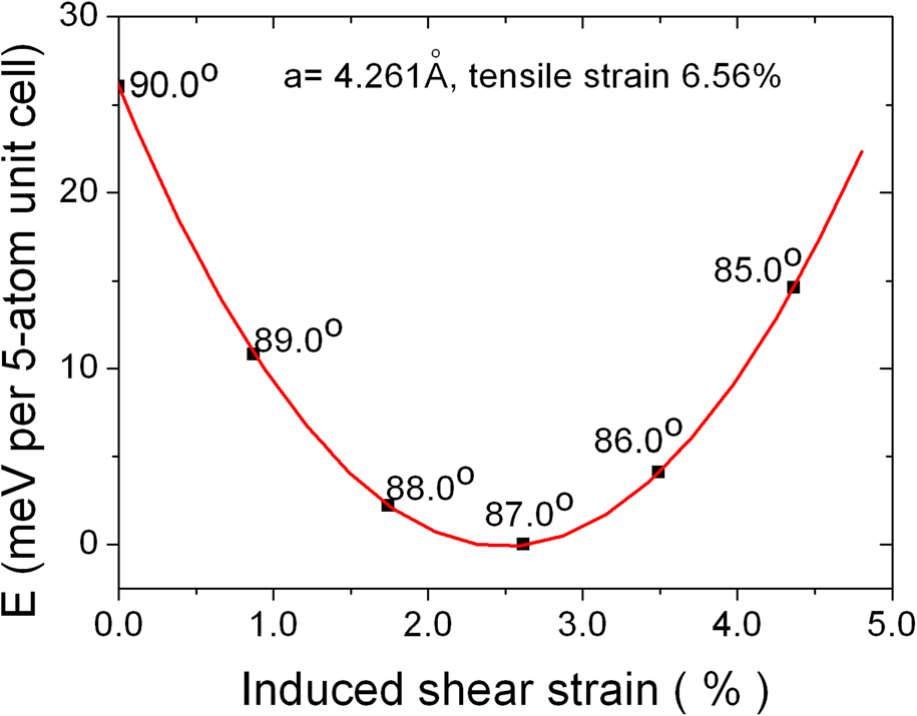
Illustration for the formation of induce deformation under tensile strain at 6.56% (i.e., *a* = 4.261Å). The most stable structure is with γ = 87.2°.

**Figure 3:**
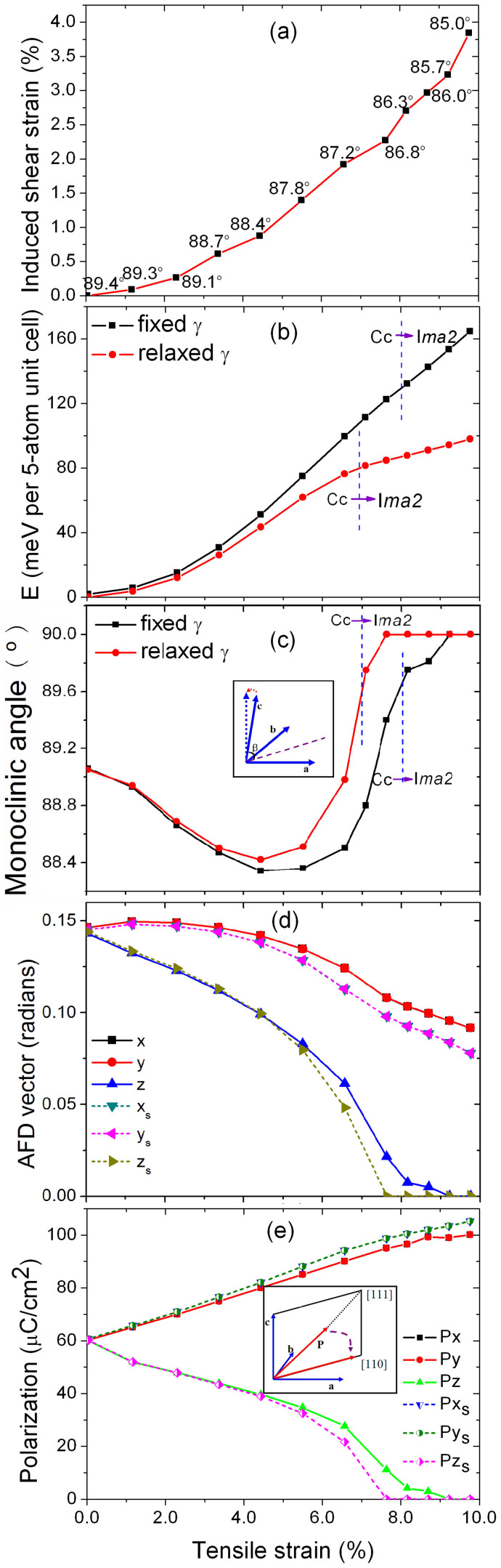
Existence of deformation under tensile strain on (001) BiFeO_3_ thin film. (a) The induced deformation – tensile strain diagram, the numbers in the figure represents the in-plane angle γ corresponding to the most stable structure under tensile strain as depicting in Figure 2; Figures 3(b)–(e) are the dependence of energy, monoclinic angle β, AFD vectors and polarization on the tensile strain of (001) BiFeO_3_ thin film with and without induction of deformation, respectively. The inset figure in Figure 3c represents the titling of monoclinic angle β. The inset figure in Figure 3d depicts the rotation of polarization vector from [111] direction (no tensile strain) to [110] direction under the tensile strain.

As mentioned above, experimental studies to date show only small deformation under ordinary conditions^28^,^29^. However, tensile strain tends to elongate lateral bonds. This very naturally leads to symmetry breaking, one possibility being deformation and another being strongly enhanced polar behavior. We studied the behavior of (001) BFO films under a tensile strain with in-plane angle, *γ* (Figure 1), fully relaxed (we note here that the in-plane angle *γ* corresponds to the most stable BFO structure under a specific tensile strain as depicted in Figure 2 for BFO at a tensile strain of ~6.6%). The relationship between the tensile strain and this shear type deformation is plotted in Figure 3a. It shows the induction of a large deformation with a strong reduction of the in-plane angle *γ* from 89.4° (at zero tensile strain) to 85.0° (at ~10% tensile strain) when the tensile strain is applied to (001) BFO thin films (note that here we take the structure with in-plane angle 89.4° instead of 90° as the reference; also note that with this definition, which is computationally convenient, changing the angle does change the area, *a*^2^sin γ, and is therefore not a pure shear deformation; the area change is, however, small for the angles that we find, n.b. sinγ ≅ 1 with γ values from 85°–90°).

Figure 3b gives the energy-tensile strain phase diagram with and without allowing the induced deformation. The existence of this deformation under applied tensile strain is clearly seen. Moreover, as seen from the energies, the effect is strong and helps to stabilize (001) BFO thin films under large tensile strain (>2%) by providing a relaxation mechanism that lowers the energy. At low tensile strain (<2%), the induced deformation is small and can be neglected. We note that the occurrence of this deformation may be related to the difficulty encountered in growing (001) BFO thin films on cubic, tetragonal substrates involving high tensile strain, such as MgO with 6.4% lattice mismatch^33^,^34^. As shown in Fig. 3b, at high levels of tensile strain, free BFO exhibits a deformation that stabilizes the material. This is via a strong deformation of the in-plane angle *γ.* In epitaxy, the substrate constrains the in-plane angle *γ* to 90°. Therefore, the constraint imposed by the substrate is energetically disfavored and thus works against the formation of (001) BFO thin films on cubic, tetragonal substrates of high tensile strain. By this we mean that the constraint that prevents deformation is unfavorable and that the growth would be more favorable under conditions that allow the deformation. In any growth, there are three main possibilities: (1) a metastable phase is nucleated, in which case strain will be relaxed by dislocations and the phase can be grown to infinite thickness, (2) an epitaxially stabilized phase is formed that can be grown up to some maximum thickness, or (3) the phase is unstable and is not readily stabilized by epitaxy at all. In the last instance, it can be that a different phase grows, i.e. not the predicted (in this case, orthorhombic) phase, or that growth attempts lead to poorly crystalized, amorphous or phase separated films. Thus in the tensile regime, epitaxy must not only constrain the two in-plane lattice parameters, which would prefer a different length, but also must constrain the angle between them, further disfavoring growth.

Without deformation, the C*c* to I*ma2* transition occurs at a tensile strain of 8% as reported by Refs. 5, 6. The occurrence of deformation shifts the C*c* to I*ma2* phase transition to a lower tensile strain at 7% (see Figure 3b). Along the z-axis, [001] direction, the oxygen octahedra slightly tilt away from each other in the deformation-free structures so that C*c* symmetry is retained. However, with deformation, the oxygen octahedra are not tilted and the structure exhibits I*ma2* symmetry. The dependence of monoclinic angle *β* (with and without deformation) on tensile strain is shown in Figure 3c, with the monoclinic angle *β* reaching 90° at a lower tensile strain due to the occurrence of deformation. This suggests that under applied tensile strain, the deformation helps prevent the monoclinic tilt of the out-of-plane lattice vector ***c***. Figure 3d gives the dependence of the antiferrodistortive (AFD, octahedral tilt/rotation) vectors on the tensile strain. Here, the direction is characterized by the axis about which the octahedral tilt and the magnetude is the angle. As tensile strain increases, the tilt/rotation becomes weaker, and this is even more so with deformation. Since the antiferromagnetic (AFM) ordering and magnetization are strongly coupled with oxygen octahedra tilt^35^, one might infer that the strain-induced shear deformation will also affect the magnetism to a certain extent. It will be of interest to study this possibility in future experiments and also detailed calculations including spin orbit and non-collinear spins.

With the understanding that a shear deformation can be induced by the application of a tensile strain to BFO thin films, the next interesting question is how this affects the ferroelectric polarization. To address this, we calculated the polarization using the Berry phase method. As shown in Figure 3e, under an appropriate tensile strain the polarization vector rotates from [111] direction to the in-plane [110] direction which is consistent with prior reports^5^,^6^. The induced deformation has a strong impact only at a high tensile strains greater than 4%, where the polarization in the [110] direction is enhanced, while the out of plane component is reduced. This behavior has also been found in other ferroelectric thin films such as BaTiO_3_ and PbTiO_3_^32^. With the induced deformation, the polarization in the [110] direction can reach a value of 140 μC/cm^2^ (P_[110]_ ~√2*P_[100]_) (We note here that in this work, we only report the results based on the assumption of G-type antiferromagnetic order for BFO structure, whereas the weak ferromagnetism due to spin canting of the two iron atoms in the unit cell is not included.).

### Influence of tensile strain on the energy band gap of (001) BiFeO_3_

**Figure 4:**
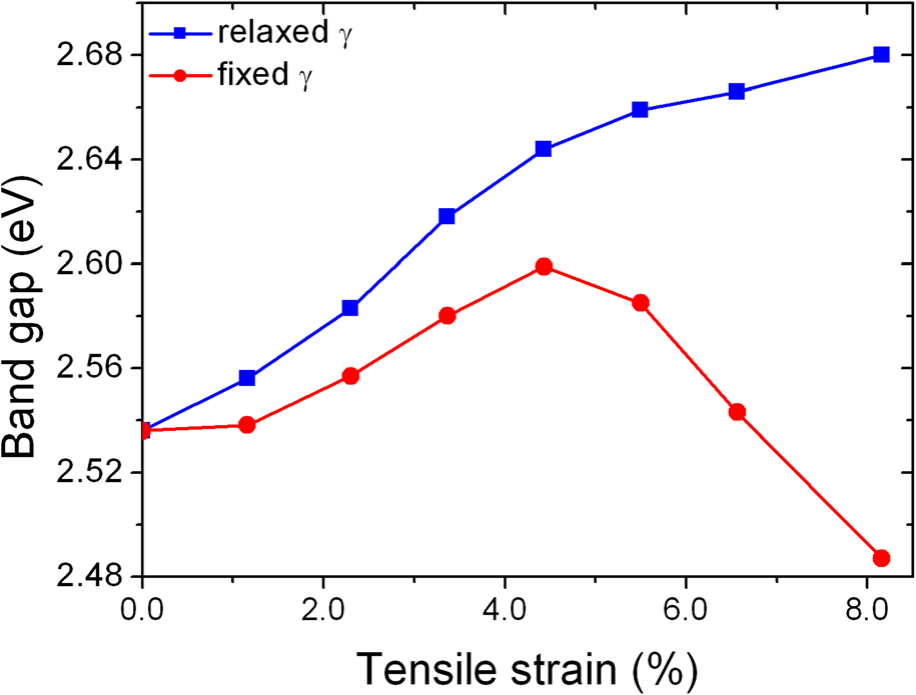
Dependence of energy band gap on the tensile strain with deformation (blue squares) and without deformation induced (red circles).

The influence of compressive strain on electronic structures and optical properties of BFO thin films has been experimentally studied, although contradictory results have been reported. The band gap of BFO was reported to be insensitive to applied strain in Ref. 36, but strongly sensitive in Ref. 37, 38. In this regard, we investigated the impact of tensile strain on the energy band gap of (001) BFO thin films. The dependence of energy band gap on tensile strain is shown in Figure 4, where one can see that the induced shear deformation leads to a strong increase in the energy gap. Thus, with the shear deformation is allowed, there is an increase in the energy gap under tensile strain. The origin of this band gap increase is readily understood. BFO is a largely ionic crystal. In such materials, lattice distortions are driven by optimization of the ionic bonding as well as covalency effects, such as the lone pair chemistry of Bi, i.e. the hybridization between nominally unoccupied Bi 6*p* orbitals and the 2*p* orbitals on neigboring O atoms. Both of these effects favor structures with higher band gaps. Specifically, the conduction bands in BFO have cation character, and therefore are pushed to higher energy when the positively charged cations adopt favorable positions from an electrostatic point of view. Moreover they have anti-bonding character with O and are also pushed up in energy by increased hybridization. Therefore, while in general there may be competing effects in perovskites, such as band width changes, it is not surprising that a symmetry lowering distortion that lowers the energy also increases the band gap in BFO. On the other hand, if shear deformation is not allowed, the energy band gap initially increases with tension and then starts to decrease when the tensile strain is larger than 4.8%. This decrease reflects the strong instability of BFO thin films under high tensile strain when it is not allowed to deform, consistent with the above argument.

### Stability of P*mc2_1_* phase of (001) BiFeO_3_ thin film under tensile strain

**Figure 5:**
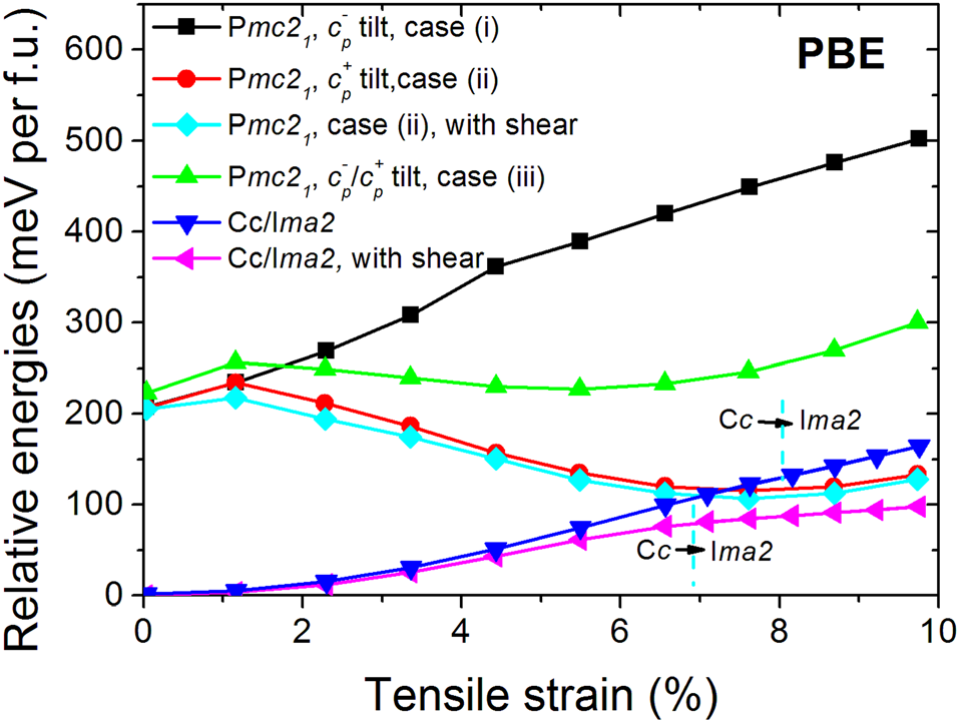
The stability of phases C*c*, I*ma2*, P*mc2_1_* with and without induced shear deformation. There are three models to describe the P*mc2_1_* symmetry of (001) BFO thin film (see main text and supporting information), case (i) anti-phase tilt **c_p_^−^**; case (ii) in-phase tilt **c_p_^+^**, NaNbO_3_ model; and case (iii) a hybrid **c_p_^−^/c_p_^+^** tilt, AgNbO_3_ model. Case (ii) results in a much lower energy structure for Pmc2_1_ symmetry. The induction of shear deformation, the pink triangle line, stabilizes the epitaxial (001) BFO thin film under tensile strain. The induced shear strain helps to further stabilize the P*mc2_1_* phase, the cyan diamond line, but it is unstable phase in comparison to Cc, I*ma2* phases.

We now turn to the phases that occur under tensile strain. It was recently reported that a phase P*mc2_1_* becomes more stable than C*c* and I*ma2* at misfit strain >5%^6^. The first question is whether the stability of the P*mc2_1_* phase will overcome the shear deformation in stablizing (001) BFO thin films at high tensile strain. To address this we studied the stability of P*mc2_1_* phase using the PBE-GGA method and also did calculations with the methods reported in Refs. 5, 6. With the epitaxial condition **a_1_** = 2a_IP_**x**, **a_2_** = 2a_IP_**y** and **a_3_** = a_IP_ (δ_1_ **x** + δ_2_ **y** + (2 + δ_3_)**z**) reported in Ref. 6, there are three structural models for describing the P*mc2_1_* phase (see supporting information for details). The first model, case (i), has one anti-phase tilt of FeO_6_ octahedra along the pseudocubic [001]_pc_ direction, **c**_pc_^−^ (a^0^b^0^c^−^). This model gives a very high energy. The second model, case (ii), has the same structure as NaNbO_3_^39^,^40^,^41^ with one in-phase tilt of the FeO_6_ octahedra along the pseudocubic [001]_pc_ direction, **c**_pc_^+^ (a^0^b^0^c^+^). This model has been theoretically applied to (001) epitaxial BFO thin films^6^ and was also studied for AgNbO_3_, where it was not found to be an appropriate model^42^. The third model, case (iii), has the AgNbO_3_ structure with a doubled unit cell along the a-axis (along the [001]_pc_ axis) having two in-phase tilts and two anti-phase tilts along the [001]_pc_ axis, i.e., **c**_pc_^+^ and **c**_pc_^−^ with antiphase tilts along the [100]_pc_ and [010]_pc_ axis, ((a^−^/a^0^), (b^−^/b^0^), (c^+^/c^−^)). The energy-misfit strain phase diagram, Figure 5, shows that the in-phase tilt model, case (ii), results in the lowest energy for P*mc2_1_* BFO thin films. However, the transition between C*c*-P*mc2_1_* takes place not at 5% as reported by Ref. 6 but at substantially larger different misfit strains, ~7.4% (see Figure 5, the cross between the red line and blue line). We did tests to understand the origin of this difference from Ref. 6 and found the origin is in the frozen core approximation. Specifically, we treated the Fe 3p semicore state as a valence state, while in Ref. 6 the Fe 3p state was treated as a core state, thereby imposing a frozen core approximation on it. We reproduced the results reported there when we use the same treatment with and without frozen core approximation (see supporting information). The results show a significant difference between these two approximations. In any case, we compare the stabilities of the C*c*, I*ma2*, and P*mc2_1_* with deformation induced C*c*, I*ma2*, and P*mc2_1_* phase using the PBE-GGA. As shown in Figure 5, the C*c* and I*ma2* (at misfit strain >7%) phase with induced deformation is still the most stable phase under tensile strain. The induced shear strain helps to further stabilize the Pmc2_1_ phase but the effect is minor in comparison to C*c* and I*ma2* phases.

## Discussion

In summary, we studied epitaxially strained (001) BFO thin films under tensile strain using first-principles calculations. Interestingly, (001) BFO thin films spontaneously deform under tensile strain. This shear deformation can stabilize the samples if the contraints do not prevent it. Our results suggest one reason for the difficulty in growing BFO under large tensile strain, specifically the inhibition of deformation by substrate clamping. That is, the normal conditions for growth involve clamping of two in-plane lattice constants, plus the angle corresponding to the deformation all of which prefer different values in this case. It will be interesting to investigate growth on miscut substrates compatible with the shear deformation both to access the physical properties of the deformed phases and also to investigate the effect on the growth conditions. We investigated into the stability of P*mc2_1_* phases including consideration of different possible tilt patterns, but we still find that the presence of the deformation makes the C*c*/Ima*2* phases more stable than all the P*mc2_1_* phases. Moreover, we studied the effects of the induced deformation on the phase transitions, monoclinic tilt, oxygen octahedra tilt, polarization and band gap of BFO thin films. The deformation shifts the C*c* to I*ma2* phase transition towards a lower tensile strain (~1% less). It improves the polarization in the [110] direction, and reduces the polarization in the [001] direction. It also strongly affects the electronic structures of BFO, especially at high tensile strain (>2%).

BFO is a remarkably versatile oxide in that its properties can be tuned by strain over an exceptionally large range. This is due to the interplay of different competing perovskite structure distortions that coexist in the material, specifically various patterns of tilt and ferroelectric distortions. Changing balances between these accommodates exceptionally large strains that couple to physical properties. The present work points to substantial deformation as an important additional structural response that is coupled to tensile strain in BFO.

## Methods

We start with the influence of deformation on the epitaxial (001) BFO thin films. We used periodic bulk cells. Thus the calculations are for strained bulk. The calculations are to model the effect of strain on BFO. Normally in experiments, this is imposed by epitaxial constraints on films. However, such films are thick enough that they are generally viewed as bulk BFO with imposed strains. We conducted first-principles calculations with the generalized gradient approximation of Perdew, Burke and Ernzerhof (PBE-GGA)^43^ as implemented in the software package VASP employing the projected augmented wave (PAW) method. A 5 × 5 × 5 Monkhorst-Pack k-mesh, and an energy cut-off of 500 eV were used. The Bi (5d^10^6s^2^6p^3^), Fe (3p^6^3d^6^4s^2^) and O (2s^2^2p^4^) are chosen as valence states. This choice was based on testing in which we employed different functionals, both with and without an additional *U* (e.g. LSDA + U, etc.) for rhombohedral BFO, where we found the best agreement with experiment for the standard PBE functional with G-type magnetic ordering. All atomic positions were fully relaxed until the final Hellmann-Feynman forces were less than 1 meV/Å. The c lattice constant was fully relaxed for each strain. The ferroelectric polarization was calculated by using the Berry phase method^44^,^45^. A fully relaxed R*3c* structure with *a_0_* = 3.90Å and α = 89.36° (which is very slightly different from the experimental bulk R*3c* structure) is chosen as the reference structure having no deformation. The normal strain and deformation are defined as *η_xx_* = *η_yy_* = (a − a_0_)/a_0_ and *η_xy_* = *η_yx_* = tan*τ* = tan((90 − γ)/2) (see Figure 1), respectively. To study the effects of deformation, we employ a ten-atom unit cell with G-type antiferromagnetic order and lattice vectors **a_1_** = (Δ_s_ + Δ_m_, a + Δ_m_, c), **a_2_** = (a + Δ_m_, Δ_s_ + Δ_m_,c), **a_3_** = (a + Δ_s_, a + Δ_s_, 0). Variation of Δ*_s_* results in a variation of deformation angle *τ* via tan*τ* = Δ_s_/a. For computational simplicity, only monoclinic phases M_A_ and M_B_ are considered with monoclinic angle *β* given by tan*β* = c/(√2Δ_m_).

To investigate the impact of tensile strain on the energy band gap of (001) BFO thin films, we used a different approach, specifically hybrid functional, PBE0^46^,^47^ calculations as implemented in the WIEN2K software package (http://www.wien2k.at/), which implements a general potential Linearized Augmented Plane Wave (LAPW) method. We used PBE0 because this functional improves the band gap of BFO, and in this regard give much better results than the PBE- GGA, which significantly underestimates the gap. In our calculations, muffin tin radii R_MT_ of 2.3, 1.9, and 1.6 a.u were chosen for Bi, Fe, and O, respectively. Inside the atomic sphere, the partial waves are expanded up to l*_max_* = 10. The number of plane waves was limited by a cut-off K*_max_* = 7.0/R_min_ (R_min_ is the minimum sphere radius R_MT_, i.e. 1.6 a.u). The interstitial charge density is Fourier-expanded with G*_max_* = 16 Ry. A 12 × 12 × 12 k-mesh in the full Brillouin zone was used. The Bi 5d and Fe 3p semi-core states were treated with the valence states using local orbital extensions. To obtain good result for the band gaps that can be directly compared with experiment, we benchmark the theoretical optical properties of rhombohedral and tetragonal BFO with experimental reports^36^,^48^. We found that good agreement with experiments is obtained when an up shift of 0.42 eV is applied. Therefore we computed the band gaps using PBE0 plus a shift of 0.42 eV. This is simply a heuristic. We used it to obtain results for the band gaps that could be directly compared with experiment. Presumably a similar result could be obtained by taking the amount of Hartree-Fock mixing as a parameter to fix the gap, as is sometimes done in hybrid functional calculations, but as mentioned, we simply used PBE0 and add a shift of 0.42 eV, which also involves adjustment of a single parameter. This was used to obtain the band gap variation with tensile strain and thus elucidates the effect of the deformation on the gap.

## Author Contributions

Theoretical calculations were carried out by Z.F., K.P.O. and M.B.S. All authors contributed to interpretation of the results. The manuscript was prepared by Z.F., J.W., A.H., D.J.S. and K.P.O.

## Supplementary Material

Supplementary Information Structural Instability of Epitaxial (001) BiFeO_3_ Thin Films under Tensile Strain
