# Supplementary material for: Structural Instability of Epitaxial (001) BiFeO3 Thin Films under Tensile Strain
Source: Sci Rep. 2014 Apr 10;4:4631. doi: 10.1038/srep04631 (PMC3982161; doi:10.1038/srep04631)
Supplement: Supplementary Information — Structural Instability of Epitaxial (001) BiFeO3 Thin Films under Tensile Strain [file srep04631-s1.doc]

Supporting Information: Structural Instability of Epitaxial (001) BiFeO_3_ Thin Films under Tensile Strain

Zhen Fan,^1^ John Wang,^1^ Michael B. Sullivan,^2^ A. Huan,^2^ David J. Singh,^3^ Khuong P. Ong^2, *^

^1^ Department of Materials Science and Engineering, National University of Singapore, 9 Engineering Drive 1, 117576, Singapore

^2^ Institute of High Performance Computing, Agency of Science, Technology and Research (A*STAR), 1 Fusionopolis Way, 138632, Singapore

^3^Materials Science and Technology Division, Oak Ridge National Laboratory, Oak Ridge, Tennessee 37831-6056, USA

* To whom correspondence should be addressed [ongpk@ihpc.a-star.edu.sg](mailto:ongpk@ihpc.a-star.edu.sg)

**Possible theoretical crystal structures of (001) epitaxial BiFeO_3_ thin film within the P*mc2_1_* symmetry**

Based on the epitaxial condition **a_1_**= 2a_IP_ **x**, **a_2_** = 2a_IP_ **y** and **a_3_** = a_IP_ (δ_1_ **x**+δ_2_ **y**+(2+δ_3_)**z**) as reported in Ref.[6] three different models for P*mc2_1_* structures can be derived depending on the tilt of FeO_6_ octahedra along the [001]_pc_ axis (the subscript pc denotes the pseudocubic unit cell):

1. One anti-phase tilt along the pseudo-cubic c_pc_-axis [001]_pc_, **c**_pc_^-^, Fig. S1
2. One in-phase tilt along the pseudo-cubic c_pc_-axis [001]_pc_, **c**_pc_^+^, NaNbO_3_ like structure, Fig. S2
3. A sequence of in-phase / anti-phase tilt along the [001]_pc_ axis (a hybrid case of (i)+(ii)), **c**_pc_^+^ /**c**_pc_^-^, AgNbO_3_ like structure, Fig. S3

These three P*mc2_1_* structures are theoretically reported in Table S1 (case (i)) and Table S2 (case (ii) and (iii)) at tensile strain of 7.6% within the PBE calculations. The lattice parameters are

- Case (i): **a** = **b** = 8.6074 Å (=2**a**_pc_), **c**= 7.3300 Å (=2**c**_pc_) $\alpha=\beta=\gamma=90^{o}$
- Case (ii): **a**=14.7800 Å (= 4**c**_pc_), **b** = **c** = 6.0854 Å (=√2 **a**_pc_) $\alpha=\beta=\gamma=90^{o}$
- Case (iii): **a**=14.9000 Å (= 4**c**_pc_), **b** = **c** = 6.0854 Å (=√2 **a**_pc_) $\alpha=\beta=\gamma=90^{o}$

For the case (ii), models (2**a**_cp_ x 2**a**_cp_ x 2**c**_cp_), (2**c**_cp_ x √2a_cp_ x √2a_cp_), and (4c_cp_ x √2a_cp_ x √2a_cp_) result in the same structure and total energy. Therefore we report the (4c_cp_ x √2a_cp_ x √2a_cp_) model to make a comparison with the in-phase/anti-phase tilt model, case (iii).

For each of given tensile strains, i.e. in-plane lattice constants **b**_O_, **c**_O_ (the subscript O denotes the orthorhombic unit cell) were fixed, and the out-of-plane lattice constant **a**_O_ (in the direction of 4**c**_pc_) was relaxed, the relaxed **a**_O_ of case (ii) and case (iii) were almost the same, by both PBE and LSDA+U calculation. Case (ii) with in-phase tilt of FeO_6_ octahedra along the [001]_pc_ results in the most stable structure in comparison to the anti-phase tilt, case (i), and a hybrid of in-phase/anti-phase tilts, case (iii).

Table S1. Atomic coordinates of BiFeO_3_ structure within the P*mc2_1_* phase, case (i) with one anti-phase tilt along the pseudo-cubic [001]_pc_ direction at the tensile strain of ~7.6% within the PBE calculation (the LSDA+U results are not shown here).

|  | case (i) | | |
| --- | --- | --- | --- |
| Site |  |  |  |
|  | x | y | z |
| Bi1 2b | 0.50000 | 0.11359 | 0.97447 |
| Bi2 2b | 0.50000 | 0.61637 | 0.97450 |
| Bi3 2a  Bi4 2a | 0.00000  0.00000 | 0.02308  0.47631 | 0.01706  0.51696 |
| Fe1 4c | 0.22817 | 0.27754 | 0.20138 |
| Fe2 4c | 0.77177 | 0.77831 | 0.20138 |
| O1 4c | 0.19874 | 0.00275 | 0.20105 |
| O2 4c | 0.27256 | 0.26349 | 0.45215 |
| O3 4c | 0.19852 | 0.50181 | 0.20008 |
| O4 4c | 0.72469 | 0.76292 | 0.45175 |
| O5 2b | 0.50000 | 0.27790 | 0.20116 |
| O6 2b | 0.50000 | 0.21727 | 0.69731 |
| O7 2a  O8 2a | 0.00000  0.00000 | 0.75880  0.25854 | 0.15747  0.15520 |

Table S2. Atomic coordinates of BiFeO_3_ structure within the P*mc2_1_* phase, case (ii) with one in-phase tilt along the pseudo-cubic [001]_pc_ direction, and case (iii), a hybrid between anti-phase tilt, case(i), and in-phase tilt, case (ii), along the pseudo-cubic [001]_pc_ direction at the tensile strain of ~7.6% within the PBE calculation (the LDA+U results are not shown here).

|  | case (ii) | | | case (iii) | | |
| --- | --- | --- | --- | --- | --- | --- |
| site |  |  |  |  |  |  |
|  | x | y | z | x | y | z |
| Bi1 4c | 0.75013 | 0.85219 | 0.35688 | 0.75164 | 0.66927 | 0.34819 |
| Bi2 2b | 0.50000 | 0.85229 | 0.35663 | 0.50000 | 0.70775 | 0.22337 |
| Bi3 2a | 0.00000 | 0.85159 | 0.35924 | 0.00000 | 0.63883 | 0.35205 |
| Fe1 4c | 0.62507 | 0.67629 | 0.78161 | 0.62660 | 0.85167 | 0.74312 |
| Fe2 4c | 0.12501 | 0.32341 | 0.28257 | 0.12802 | 0.18089 | 0.28464 |
| O1 4c | 0.75012 | 0.78539 | 0.71687 | 0.74538 | 0.68970 | 0.71897 |
| O2 2b | 0.50000 | 0.78523 | 0.71636 | 0.50000 | 0.92408 | 0.78751 |
| O3 4c | 0.62524 | 0.55083 | 0.48391 | 0.60265 | 0.63043 | 0.47739 |
| O4 4c | 0.62499 | 0.04132 | 0.47141 | 0.65272 | 0.08939 | 0.55300 |
| O5 2a | 0.00000 | 0.21477 | 0.21636 | 0.00000 | 0.27058 | 0.21093 |
| O6 4c | 0.12508 | 0.95869 | 0.97345 | 0.13534 | 0.04757 | 0.98768 |
| O7 4c | 0.12477 | 0.55109 | 0.48409 | 0.12121 | 0.45563 | 0.47650 |


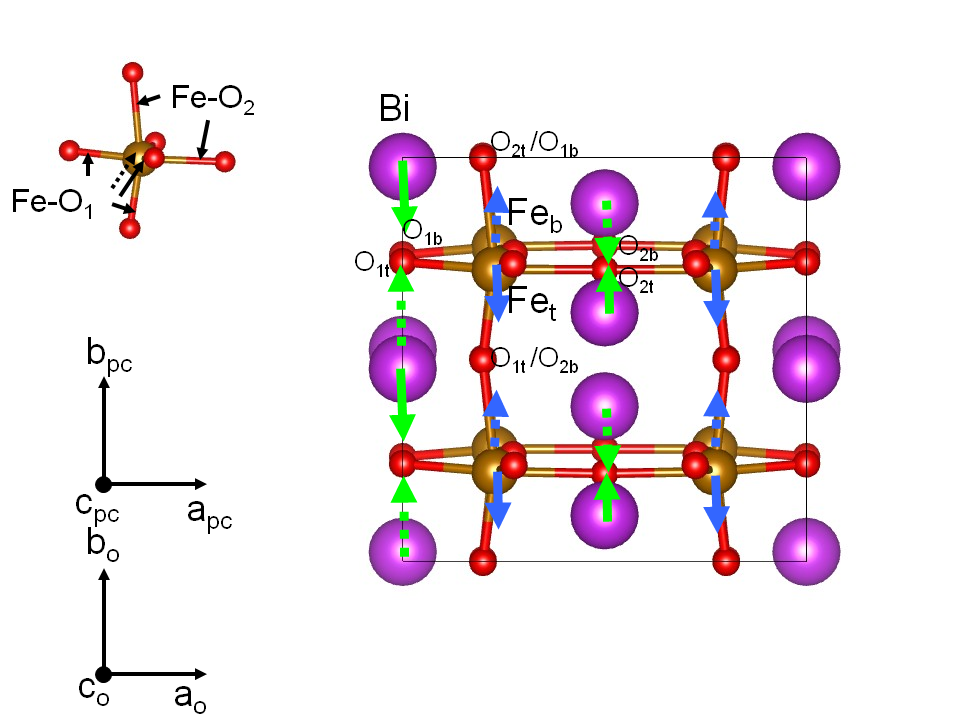


**Figure S1**. (color online) Projection of the P*mc2_1_* structure, case (i), on (001)_pc_ plane. The anti-phase **c**_pc_^-^ tilts can be seen from the rotation of the Fe_t_-O_1t_ bond relative to the Fe_b_-O_1b_ bond. Both Bi and Fe atoms move towards to positive [010]_pc_ and negative [010]_pc_ direction, respectively (solid arrows show the movement of atoms in the top layer, while dotted arrows show the movement of atoms in the bottom layer). The oxygen octahedral has two long bonds, Fe-O_2_, ~ 2.3 Å, and four short bonds, Fe-O_1_, 1.9~2.0 Å. The sequences of the arrangement of long and short Fe-O bonds along [010]_pc_ direction are different for top and bottom layers leading to different movements of Fe atoms along [010]_pc_ direction.


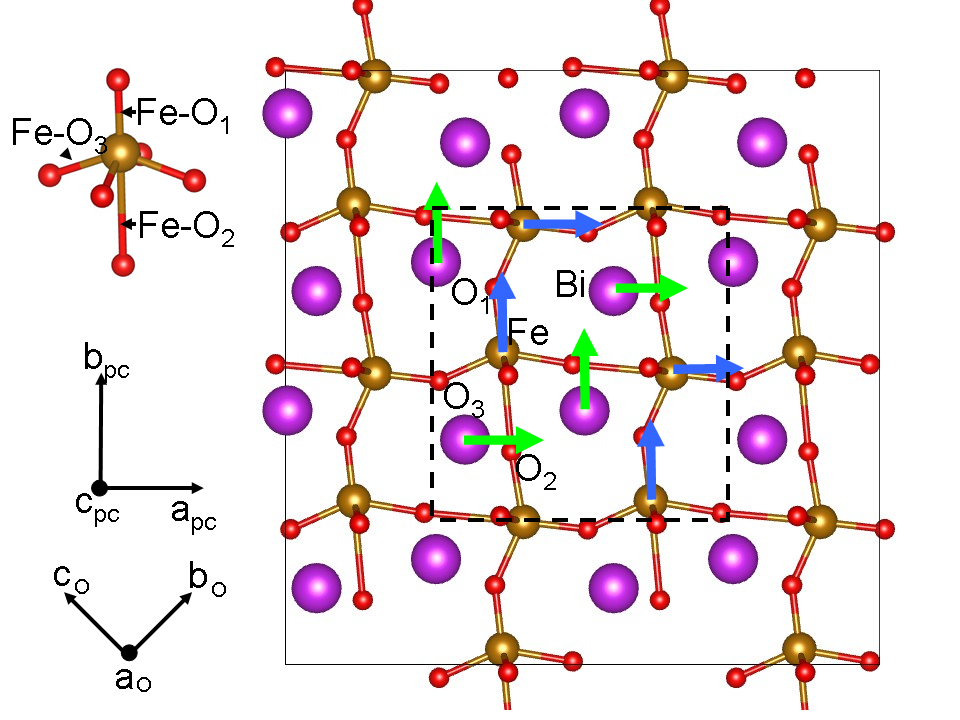


**Figure S2**. (color online) Projection of the P*mc2_1_* structure, case (ii), on the (001)_pc_ plane. Only in-phase **c**_pc_^+^ tilt of oxygen octahedra is observed. The dotted line square shows a checkerboard pattern of the displacements of Bi and Fe atoms along [100]_pc_ or [010]_pc_ direction. There is only one type of oxygen octahedral, which is highly similar to the one found in giant tetragonal BFO phase. The structure has one long Fe-O bond (Fe-O_2_ ~2.9 Å), one short Fe-O bond (Fe-O_1_ < 1.9 Å) and four equatorial Fe-O bonds with a relatively medium length (Fe-O_3_ ~2.0 Å).


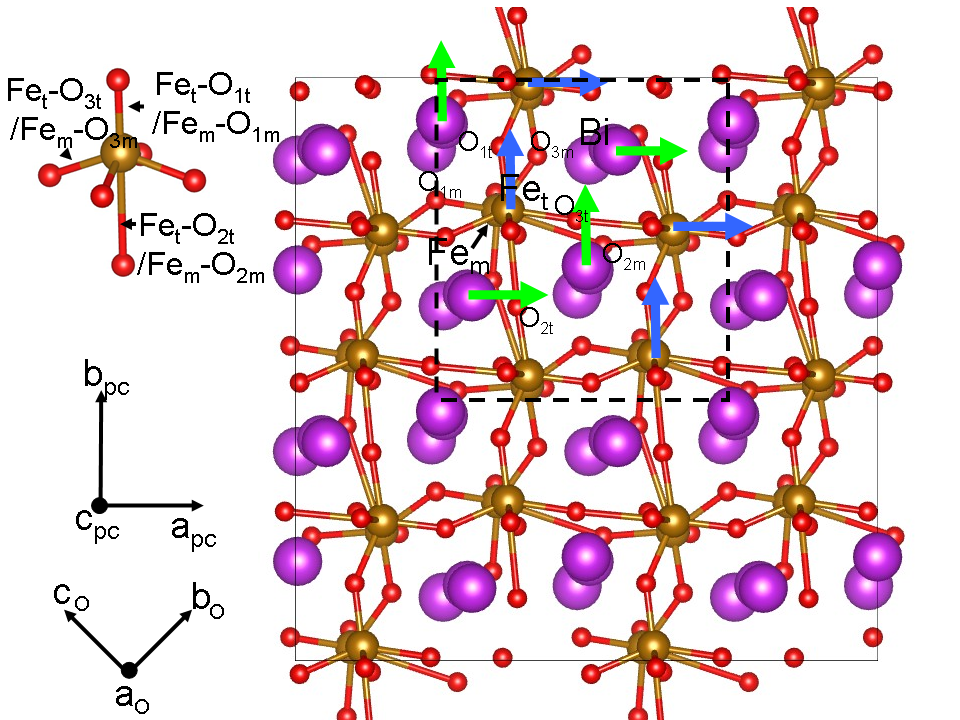


**Figure S3**. (color online) Projection of the P*mc2_1_* structure, case (iii), on the (001)_pc_ plane. A sequence of the in-phase **c**_pc_^+^ tilt and the anti-phase **c**_pc_^-^ tilt is formed along the **c**_pc_ direction. The dotted line square show the checkerboard pattern of the displacements of Bi and Fe atoms along [100]_pc_ or [010]_pc_ direction. There are two types of oxygen octahedral, the first is located at the top/ bottom layer having a long Fe-O bond (Fe_t_-O_2t_ bond) with the length of ~2.9 Å, the other is at two middle layers having even larger bond length (Fe_m_-O_2m_ bond) of 3.3 Å.

To understand the difference between our theoretical results with what were reported by Yang et al. [6], we conducted a LSDA+U (U=3.8eV as used by Ref. [6]) calculations with pseudo-potential of Fe having the electronic configuration of (3d^7^4s^1^), frozen core, and (3p^6^3d^6^4s^2^), unfrozen core. The obtained results for the first case are in perfect agreement with Ref. [6], see Fig.S4a, saying that the Fe 3p^6^ electrons have been frozen by Yang et al. [6] (see the main text for more detail). The results for the second case within different Pmc2_1_ symmetries, c^-^_p_ tilt, c^+^_p_ tilt, c^-^_p_/c^+^_p_ tilt, (see main text for more detail) show a substantial shift of Cc-P*mc2_1_* transition to higher tensile strain, 6.5% in comparison to 5% as reported by Yang et al [6].


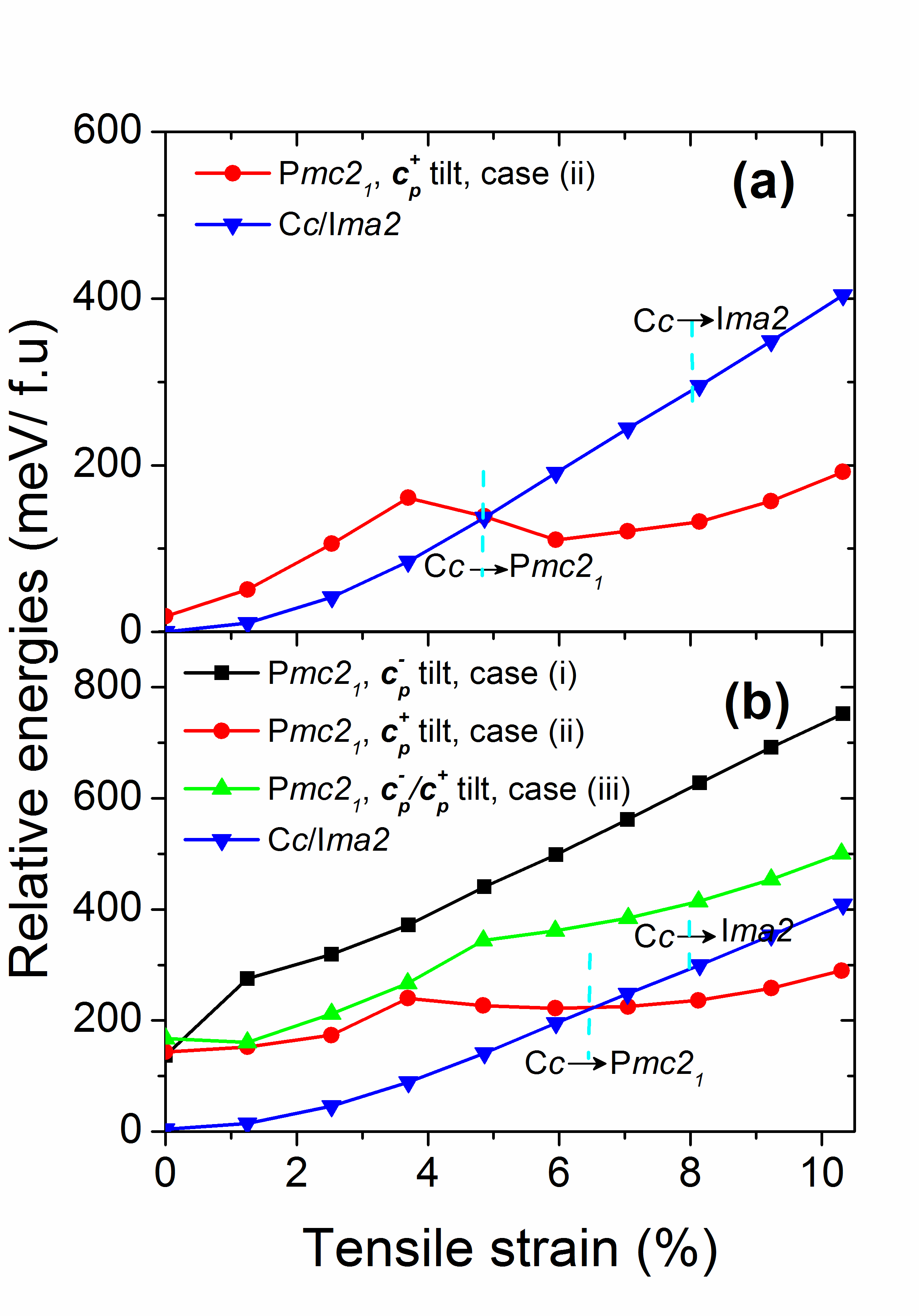


**Figure S4.** (color online) The energy-misfit strain phase diagram of epitaxial (001) BiFeO_3_ thin film within different symmetries, C*c*, I*ma2*, and P*mc2_1_*. Here the potential of Fe has only 8 valence electrons (3d^7^4s^1^) with Fe-3p electrons are frozen. The results are in well agreement with report by Yang et al, Ref.[6] but it is very much different with the un-frozen Fe -3p electrons potential with 14 Fe-electrons in the valence band (3p^6^3d^6^4s^2^), (see Fig. S4b).
